# Phenotypic variation in populations of the mosquito vector, *Aedes aegypti,* and implications for predicting the effects of temperature and climate change on dengue transmission

**DOI:** 10.1371/journal.pntd.0013623

**Published:** 2025-11-18

**Authors:** Nina L. Dennington, Marissa K. Grossman, Janet L. Teeple, Leah R. Johnson, Marta S. Shocket, Elizabeth A. McGraw, Matthew B. Thomas

**Affiliations:** 1 Department of Entomology, The Pennsylvania State University, University Park, Pennsylvania, United States of America; 2 The Center for Infectious Disease Dynamics, The Huck Life Sciences, The Pennsylvania State University, University Park, Pennsylvania, United States of America; 3 Department of Statistics, Virginia Tech, Blacksburg, Virginia, United States of America; 4 Department of Geography, University of Florida, Gainesville, Florida, United States of America; 5 Lancaster Environment Centre, Lancaster University, Lancaster, United Kingdom; 6 Department of Biology, The Pennsylvania State University, University Park, Pennsylvania, United States of America; 7 Department of Entomology & Nematology, University of Florida, Gainesville, Florida, United States of America; 8 Department of Biology, University of York, York, United Kingdom; Institut Pasteur, FRANCE

## Abstract

There is concern that increases in temperature due to climate change could lead to shifts in the transmission dynamics and distribution of mosquito vectors. Many current models assume there are ‘average’ thermal performance curves for a given vector species’ life-history traits. However, this ‘one-size-fits-all’ assumption ignores the potential for standing phenotypic variation in life-history traits to create population-specific differences in thermal performance. In this study, we explored thermal performance of five independent field populations of *Ae. aegypti* from Mexico, together with a standard laboratory strain. We reared these six populations at temperatures between 13°C- 37°C to generate thermal performance curves for a suite of life-history traits. Composite models integrating these traits revealed the effects of temperature on population growth rates and dengue virus transmission potential. The results provide strong evidence for the potential for local adaptation in *Ae. aegypti* populations, challenging the applicability of ‘one-size-fits-all’ thermal performance models to assess climate impact on mosquito-borne diseases.

## Author summary

How climate affects the dynamics and distribution of mosquito-borne diseases is of considerable public health relevance, especially in a warming climate. Models often assume the relationship between temperature and transmission is fixed for mosquito species and can be extrapolated over time and space. We challenge this assumption with evidence for standing variation in populations of *Aedes* mosquitoes, the primary vectors of dengue, Zika, and chikungunya viruses. We show variation in thermal sensitivity of life-history traits for five field populations of mosquitoes and one laboratory-adapted population across 11 temperatures. We then use mechanistic models which integrate these life-history traits to understand the potential for differences in fitness and transmission for mosquito populations. Such effects will increase variation in the expected impact of climate and challenge the utility of ‘one-size-fits-all’ models to predict the effects of climate change on vector-borne disease transmission.

## Introduction

Dengue is the most widespread vector-borne disease of humans, with more than 3.9 billion people estimated to be at risk in over 128 countries [1]. Dengue virus is transmitted by mosquitoes, with *Aedes aegypti* as the principal vector [2]. Transmission of dengue virus is strongly influenced by temperature, humidity, rainfall along with other climate factors [2–5] and as such, there is substantial interest in the extent to which transmission will be affected by climate change [6]. Numerous studies suggest a likely net increase in the overall size of the population at risk of dengue and other arboviruses in the coming decades [7–14].

The climate-sensitivity of mosquito-borne diseases derives from the fact that many mosquito and pathogen life-history traits are strongly affected by temperature. Traits of many ectotherms are temperature-dependent, often exhibiting nonlinear thermal performance curves [15–17]. For mosquitoes and their pathogens, these traits include both fitness traits, such as larval survival, development rate, adult survival, fecundity, vector competence, and pathogen development rate, as well as behavioral traits such as biting rate, mating and foraging behaviors [18–29]. Additionally, these traits show variation between individuals depending on nutritional status, body size, and genetics [30–33].

Thermal performance curves provide a framework to assess how environmental temperature affects biochemical and physiological processes. In order to understand how changes in vital rates impact transmission, it is necessary to integrate individual traits into temperature-dependent fitness metrics such as the Basic Reproduction Rate (R_0_, defined as the number of secondary cases resulting from an initial primary case introduced into a population of susceptible hosts), or vectorial capacity (a measure of the transmission potential of a mosquito population defined as the number of potentially infectious bites that would eventually arise from all the mosquitoes biting an infectious host on a single day). These temperature-dependent transmission metrics have been used extensively to investigate variation in the relative environmental suitability for transmission over time and space [7–11,19,27,34–41].

Common to nearly all mechanistic models that follow the thermal performance-R_0_ approach, is the assumption that thermal performance curves are fixed for a given mosquito species and associated pathogen. However, this assumption ignores the potential for the environment to impact thermal performance curves at the local population level [42]. Local adaptation can occur when there is spatial variation in selection due to interactions with the environment leading to a relative increase in fitness at a local level [42]. The evidence for local thermal adaptation in insects is mixed. Some studies indicate the potential for populations to shift thermal performance curves through evolutionary or plastic responses [43–48], while others suggest limited adaptation in response to variation in climate [49–52]. Additionally, it is observed in insects that there is greater potential for local adaptation at cold temperatures, though observed non-lethal adaptation to warmer temperatures is possible [53–55]. In general, it is expected that short generation times and high intrinsic population growth rates, both of which are true for mosquito vectors, should increase the probability of adaptation to changing conditions [56,57]. Local adaptation in mosquitoes to other environmental drivers, such as insecticide exposure, humidity, or daylength, support this suggestion [57–63].

Common garden experiments, in which populations collected from distinct geographic locations are examined together under shared conditions, have been used to compare thermal performance of different insect populations collected across environmental gradients and isolate differences between populations due to genetic variation [31,64–68]. Common garden experiments are designed to study the adaptive genetic variation among populations when stripped of environmental influence [67,69,70]. The rationale behind the experimental design is to understand the difference in the genetic basis of complex traits by reducing effects of genotype-by-environment interactions along with phenotypic plasticity (*Text A in* *S1 Appendix*) and as such are often used to test for signals of local adaptation [71–73]. Recently, we followed this approach to demonstrate differences in thermal tolerance (measured as knockdown rate in response to exposure to stressful high temperature) between five field-derived populations of *Ae. aegypti* from different locations in Mexico, together with a longstanding lab colony [47]. These results were strongly suggestive of local adaptation, but knockdown rate does not describe overall fitness, nor enable us to explore possible implications for transmission. The aim of the current study, therefore, is to extend research in this system to characterize thermal performance of a suite of life-history traits and determine whether there are between-population differences in thermal dependence of mosquito fitness and dengue transmission potential. We find strong evidence for genetic differences and local adaptation, challenging the utility of ‘one-size-fits-all’ thermal performance models to capture the spatial or temporal influence of temperature and temperature change on transmission risk.

## Materials and methods

### Ethics statement

All experiments were conducted under Penn State IBC protocol # 48219. Mosquito populations were imported under CDC import permit # 2018-03-181.

### Mosquito Collection

*Aedes aegypti* mosquitoes were collected from the field in five different locations in Mexico (Cabo San Lucas, Acapulco, Monterrey, Ciudad Juárez, and Jojutla) using ovitraps. Populations from Mexico were founded with at least 100 females and to decrease the influence of causal maternal effects [74,75], mosquitoes were reared in the lab for at least one generation in standard laboratory conditions (27°C, 80% humidity, 12:12hr photoperiod) prior to experimentation. Two populations, Jojutla and Ciudad Juárez, were reared for an additional generation to ensure a large enough population for subsequent experiments. The field locations were chosen to capture a gradient of the climate and landscape (see Table A in S1 Appendix). The field populations were compared to a laboratory population (Rockefeller strain) that were maintained at Penn State University under standard insectary conditions (as described above) over many years [76].

### Experiments to generate temperature-dependent data

Mosquito life-history traits including egg-to-adult survival, mosquito development rate (or the inverse of time to adult stage), mean adult survival, fecundity, and biting rate were measured in mosquitoes reared at 13°C, 15°C, 19°C, 23°C, 25°C, 27°C, 29°C, 31°C, 33°C, 35°C, 37°C, each ± 0.2°C and 80 ± 10% relative humidity in environmentally controlled incubators with conditions monitored by data loggers [77]. These life-history measurements were replicated three times at each temperature for each population. We began with eggs from the five field populations and one laboratory line that were hatched at 27°C for 24 hours. Then, 200 first instar larvae were put into 1.89 L containers with 1 L of deionized water and 0.20 mg of larvae bovine liver powder (MP Biomedicals) and each of the three replicates were placed in the incubator at their respective temperatures. We fed larvae 0.20 mg of liver powder per larvae every other day until pupation, but once pupation began we scaled their food to the remaining number of larvae. Pupae, both living and dead, were removed and counted on the day of pupation and placed in a small cup (30 mL) with water from their original environment to allow for eclosion. Pupae were then added to a small cage (17.5 cm^3^) with continuous access to 10% sugar solution (dextrose anhydrous and deionized water). We counted the number of adults that eclosed every day. We measured mosquito development rate and egg-to adult survival for 200 individuals for every replicate at each temperature for each population. After 95% of surviving females emerged, we blood-fed females after 3–5 days using an artificial feeder (Hemotek). We used blood from de-identified human donors (BioIVT, Corp.) and so Institutional Review Boards approval and human subjects’ approval was not needed. We immediately counted the total number of blood-fed females and placed up to 10 individual females into separate containers (50 mL polypropylene centrifuge tubes) that were lined with filter paper and 7 mL deionized water to measure individual fecundity. We also placed up to 20 females into two small cages (10 in each) with a small filter paper for egg laying and to monitor adult survival (measuring fecundity for up to 30 females). We recorded the day that females in individual containers first laid eggs, which we used for fecundity measures and to approximate the biting rate (1/gonotrophic cycle length), after which we removed them from their containers and placed them into the group cages (Fig A in S1 Appendix). We extracted the water from the containers to let the filter paper dry in their respective incubators and then we counted the number of eggs from individual mosquitoes. For the course of the experiment, we offered each cage a blood meal every 4 days and counted the number of adults (both male and female) that died every day (up to 200 individuals per replicate per treatment). All treatment groups were provided equal access to blood meals and mating opportunities, ensuring that any behavioral differences influencing longevity were inherently accounted for. We censored this experiment 4 weeks after the first egg lay at each temperature. Methods used in the current study closely follow those outlined in Dennington et al. 2024 [47].

### Models of thermal performance curves

To analyze the thermal performance curve data we used a Bayesian approach following methods described in Johnson et al. (2015). In some cases, we fit multiple thermal response functions to each trait including quadratic, Brière, Poisson and binomial distributions, depending on what was appropriate given the data [36]. We fit a symmetric thermal response function to our data for fecundity (number of eggs per female for the first gonotrophic cycle) and egg-to-adult survival using a quadratic equation:


$$f(T)=\left\{ \begin{array}{c} -c\left(T{-T}_{\text{0}}\right)\left(T{-T}_{m}\right)\text{if}T_{\text{0}}\leq \text{T}\leq T_{m}\\ \text{0}\text{otherwise} \end{array}\right.$$

Mosquito development rate and biting rate were described by a Brière function:


$$f(T)=\left\{ \begin{array}{c} -cT\left(T{-T}_{\text{0}}\right)(\sqrt{T_{m}-T})\text{if}T_{\text{0}}\leq \text{T}\leq T_{m}\\ \text{0}\text{otherwise} \end{array}\right.$$

where T_0_ is the thermal minimum (below which the function is zero), T_m_ is the thermal maximum, and c is a positive constant that controls the curvature of the function (and thus the height of the curve for a given value of T_0_ and T_m_). Blood feeding rate was approximated as the reciprocal of the duration of the first gonotrophic cycle, a standard assumption used in many studies [10,78–80].

We assume that observed values for fecundity, mosquito development rate, and biting rate must be non-negative, and are thus modeled with a truncated normal likelihood:


$$F\sim TN(\mu =f(T),\sigma^{\text{2}},\text{0},\infty )$$

We chose priors for T_0_ and T_m_ to restrict each trait to its biologically realistic range, assuming temperatures below 0°C and above 45°C were fatal as previously measured [10,11,36,37]. For other parameters we chose relatively uninformative priors (Text B in S1 Appendix).

In contrast to fecundity, biting rate, and development rate, egg-to-adult survival is a probability and must be constrained to lie between 0 and 1. Thus, we modeled these data as being binomially distributed:


$$Y_{i}\sim Bin\left(p=f\left(T_{i}\right),n_{i}\right)$$

where *n* is the number of total observations of which Y were successes (i.e., survival to become adults) and the probability of a success at a particular temp, *p,* depends on temperature (f(*T*)), specifically being a piecewise quadratic, as above, but being constrained to be less than or equal than 1.

We also measured survival times (life spans) for adult mosquitos by counting the number of mosquitoes that died each day. Time to death for adult mosquitoes in each treatment were observed until a censoring time $T_{\text{cut}}$; i.e., for each temperature, survival observations were censored 28 days after the first egg lay. Thus, at each temperature for each population most mosquitoes were observed until they died, but a portion of them were still alive at the end of the 28 days, and so the survival times were right-censored. We used a variation of a Bayesian Weibull survival model. We assume that the observed lifetimes, $y_{i}$, at some particular temperature are drawn from a Weibull distribution with rate parameter β and shape parameter k, with pdf,


$$f(y;\beta ,k)=\beta k(\beta y{)}^{k-\text{1}}e^{-(\beta y{)}^{k}},$$

and corresponding CDF notated as F(y;β,k). The median, m, of the Weibull is defined as


$$m=\frac{{\left(ln\text{2}\right)}^{\text{1}/k}}{\beta }$$

which, can be rearranged to solve for β. For interpretability, we assume that the median lifetime decays exponentially with temperature across the experimental temperature range studied, that is,


ln(m)=a−bT

so that the median thermal performance curve, g(T), is


m=g(T)=exp(a−bT)

where T is the temperature, and a and b are parameters to be estimated.

The likelihood for the survival data is comprised of two components, one describing the individuals that were observed to die within the study period ($\delta_{i}=\text{1}$), and those who are censored (i.e., that survive the study period, $\delta_{i}=\text{0}$). Thus, the likelihood is given by


$$\begin{array}{c} L=\prod_{\text{i}}^{\text{n}}{\left[\text{f}\left({\text{y}}_{\text{i}};\beta ,\text{k}\right)\right]}^{\delta_{i}}{\left[F\left(y_{i}=T_{{\text{cut}}_{i}};\beta ,k\right)\right]}^{\text{1}-\delta_{i}}, \end{array}$$

where the rate parameter is defined in terms of the median thermal performance curve, $\beta =\frac{{\left(\text{ln}\text{2}\right)}^{\text{1}/k}}{g(T)}$.

We fit all models described above using Markov Chain Monte Carlo (MCMC) sampling that is implemented in JAGS, using the R package *R2jags* [81–83]. For each life-history trait or thermal response, we ran five MCMC chains with a 5,000-step burn-in and saved the subsequent 10,000 steps. We thinned the posterior samples by saving every eighth sample, for a total of 3125 posterior samples of parameters. We used these posterior samples to produce samples from the posterior distribution of each trait across temperature. We then summarize the relationship between temperature and each trait by calculating the mean and 95% highest posterior density interval (HPD interval) for the function across temperature. The HPD interval is a type of credible interval that includes the smallest continuous range containing 95% of the probability, which is implemented in the coda package [84].

### Mosquito Fitness and Transmission Calculations

To characterize overall fitness, we used a temperature-dependent model for population growth $r(T_{i})$ as previously described in Amaraesekare and Savage [85]:


$$r(T_{i})=-\mu (T_{i})+MDR(T_{i})W\left(\frac{E(T_{i})e^{\mu (T_{i})-\frac{\mu_{j}(T_{i})}{MDR(T_{i})}}}{MDR(T_{i})}\right)$$ (1)

Here MDR is mosquito development rate, E is eggs per female measured at the first gonotrophic cycle, μ is adult mortality rate and $\mu_{j}$ is juvenile mortality rate (Fig B and Table G in S1 Appendix). W(x) is the upper branch of the Lambert function. We combined the thermal performance curves for each trait to calculate temperature-dependent fitness, $r(T_{i})$, creating a unimodal curve. We followed Amarasekare and Savage [85] and truncated the results at r(Ti) = 0 rather than allowing them to go to negative values.

We estimated the posterior distribution of $r(T_{i})$ and used it to calculate the key temperature values for relative temperature dependent population fitness. We used the mean and 95% credible intervals (95% CI) for the critical thermal minimum, maximum and optimum temperatures for population fitness.

We used temperature-dependent models of transmission to investigate the potential implications for transmission of dengue virus, utilizing a previously published framework [10,11,36,37]. This approach models transmission rate as the basic reproduction rate R_0_, which is defined as the number of secondary infections originating from an initial infection when introduced to a completely susceptible population. The temperature dependent R_0_ is given by:


$${\text{R}}_{\text{0}}(\text{T})={\left(\frac{{a(T)}^{\text{2}}b(T)c(T)e^{-\frac{\mu (T)}{PDR(T)}}EFD(T)\rho_{EA}(T)MDR(T)}{{Nr\mu (T)}^{\text{3}}}\right)}^{\text{1}/\text{2}}$$ (2)

In this equation, (T) indicates that the trait is a function of temperature, a is the biting rate per mosquito, b is the proportion of infectious bites that infect susceptible humans, c is the proportion of bites on infected humans that infect uninfected mosquitoes (*b* × *c* = vector competence), μ is the adult mosquito mortality rate, PDR is the pathogen development rate (the inverse of the extrinsic incubation period), EFD is the number of eggs a female produces each day, $\rho_{EA}$ is the survival probability of mosquitoes from egg to adult, MDR is the mosquito development rate (the inverse of the egg-to-adult development time), N is the density of humans, and r is the human recovery rate.

To use this metric in this study, we have to make two approximations to connect the data and fits obtained from our empirical study to this expression. The first is in the adult mosquito mortality rate. In the original derivation of R_0_, it is assumed that mosquito lifetimes are exponential distributed with rate parameter μ. Thus, under this assumption, the mean lifetime, λ, is 1/μ, and the median lifetime, (denoted at *m* above), would be equal to $\frac{\text{ln}(\text{2})}{\mu }$. Thus, everywhere in the R_0_ equation we let $\mu =\frac{\text{ln}(\text{2})}{m}$. Similarly, we must approximate the mosquito development rate by taking the 1/ mosquito development time to get the rate of development.

In the current study we did not examine infected mosquitoes and so we used previously published thermal performance curves from Mordecai et al. (2017) for vector competence and pathogen development rate for dengue virus in *Ae. aegypti* [10]. Also, because we have no measures of human density or recovery rate we follow the approach of Mordecai et al. (2017) and normalize the R_0_ curves to a scale of 0–1. This measure of Relative R_0_ enables comparison of key features of thermal dependency including the critical minimum temperature (CTmin), the critical maximum temperature (CTmax), the optimum temperature (Topt) and the thermal breadth (Tbreadth), defined as the range where performance is above 80% optimal [55,86] but does not provide estimates of maximum performance (Pmax).

### Testing for differences between populations

We used DIC (Deviance information criterion) to test for statistically significant differences between the thermal performance curves of different populations as previously described [87–89]. Specifically, for each trait, we compared the DIC of a global model fit to the data from all populations to the sum of DIC scores from the models fit separately to data from each population (equivalent to fitting a single model assuming all parameters may vary between populations). Populations were considered significantly different for a given trait if the sum of DIC scores from the separate models was >= 2 DIC units lower than the DIC score from the global model.

## Results

**Fig 1:**
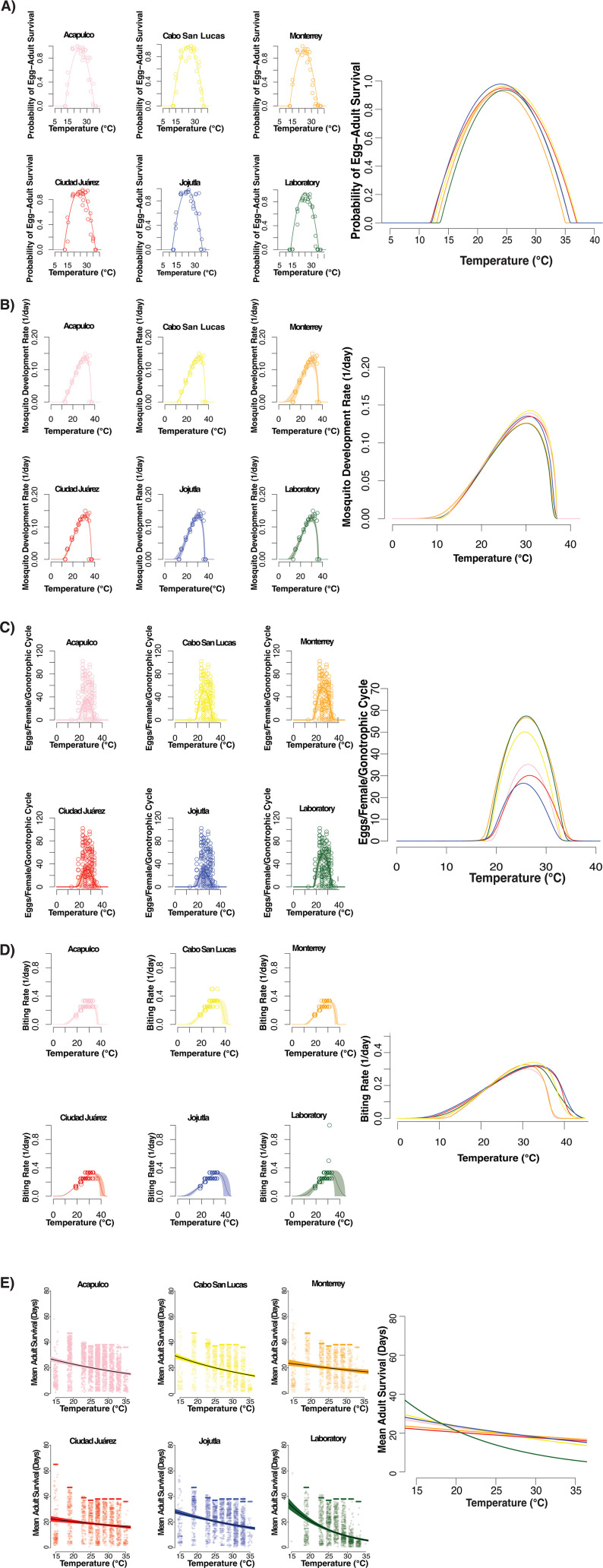
Life history traits measured for *Ae. aegypti* mosquitoes reared at 13°C, 15°C, 19°C, 23°C, 25°C, 27°C, 29°C, 31°C, 33°C, 35°C, 37°C. We measured life history traits for five populations from Mexico (Cabo San Lucas (yellow), Acapulco (pink), Monterrey (orange), Ciudad Juárez (red), and Jojutla (blue)) compared to a laboratory adapted line (shown in green). The data points for figures are the values from three replicates for each corresponding population at each temperature with individual values for adult survival and fecundity along with mean values for each replicate for biting rate, mosquito development rate and egg-to-adult survival probability. Thermal performance curves for life-history traits for the six populations were fit using Bayesian inference with weakly informative priors are shown (Model values can be found in Tables B-F in S1 Appendix). Each mosquito line was tested between 13°C-37°C, but individuals did not survive and reproduce at 13°C and 37°C. **A)** Egg-to-adult survival measured as the probability of individuals surviving to the adult stage. **B)** Mosquito development rate is the inverse of the amount of time that it takes to reach the adult stage. **C)** Fecundity is measured as individual egg production for the first gonotrophic cycle. **D)** Model fits for approximated biting rate (1/gonotrophic cycle length). **E)** Model fits for estimated mean adult survival (in days) derived from measures of daily survival of adults over 28 days post initial blood meal, censored individuals are clustered at the last day tested.

The estimated thermal performance curves for egg-to-adult survival probability, mosquito development rate, fecundity, biting rate, and mean adult survival indicated by both the empirical data and the Bayesian model fits are presented in Fig 1A-E, with the key features of these curves (i.e., the thermal optimal temperature (Topt), the maximal performance (Pmax), the critical thermal minimum (CTmin), critical thermal maximum (CTmax) and thermal breadth (Tbreadth) summarized in Tables B-F in S1 Appendix.

The between-population differences in the thermal performance curves for the juvenile traits of egg-to-adult survival and development rate were relatively small, with 1°C or less for the differences between the respective CTmin, CTmax, Tbreadth values of the different populations, although still significant according to the Deviance Information Criterion (DIC) (Fig 1A and 1B and S2-S6 Tables in S1 Appendix). For the adult traits of fecundity, survival and biting rate, the differences were more marked. Specifically, for eggs per female, there was an approximate 2°C difference between the lowest (Monterrey population) and highest (Acapulco population) values for CTmin, and 3°C difference between lowest (Jojutla population) and highest (Monterrey population) values for CTmax and clear variance between populations (Fig 1C). For the biting rate approximation, there was an approximate 6.6°C difference between the lowest (Jojutla population) and highest (Monterrey population) values for CTmin, and 4.5°C difference between lowest (Monterrey population) and highest (Jojutla population) values for CTmax (Fig 1D). For adult survival, the data did not exhibit a clear unimodal pattern across the temperature range studied so it is not possible to define values for CTmin and CTmax (Fig 1E). Nonetheless, the thermal performance models indicate significant differences between populations according to the DIC criteria. For the field strains, the population from Ciudad Juárez survived the worst at lowest temperatures while the population from Cabo San Lucas survived the best (highest median survival). Alternatively, the population from Cabo San Lucas survived the worst with the lowest median survival at high temperatures while the population from Monterrey survived the best with the highest median survival. Notably, the longstanding lab strain exhibited the most pronounced thermal response, with the highest survival at cool temperatures and the lowest survival at warm temperatures.

**Fig 2:**
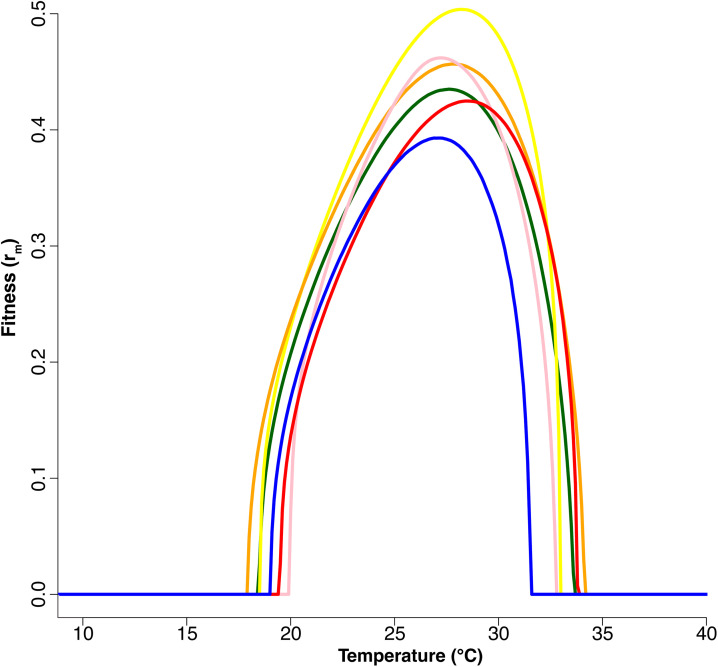
Temperature-dependent fitness (intrinsic rate of increase, r_m_) derived from individual life-history traits measured for *Ae. aegypti* mosquitoes reared at 13°C, 15°C, 19°C, 23°C, 25°C, 27°C, 29°C, 31°C, 33°C, 35°C, 37°C. We measured life history traits for five populations from Mexico (Cabo San Lucas (yellow), Acapulco (pink), Monterrey (orange), Ciudad Juárez (red), and Jojutla (blue)) compared to a laboratory-adapted line (shown in green). The lines indicate mean model fits. Fits for individual populations that include the credible intervals and values are presented in Fig C and Table H in S1 Appendix.

**Fig 3:**
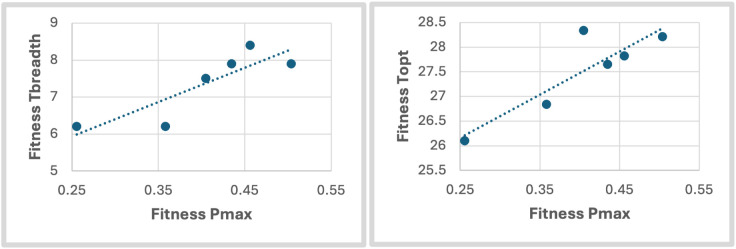
Summarized values for thermal performance curves for each population showing the relationship between (a) breadth of the curve (Tbreadth) and maximum performance (Pmax). Pearson’s correlation indicates significant strong positive relationship between Pmax and Tbreadth (r = 0.882) and Pmax and Topt (r = 0.861).

The combined effects of variation in individual traits are shown in the thermal performance curves for overall mosquito fitness, r_m_ (Fig 2). Significant differences in the DIC of the individual thermal performance curves translate to significant differences in overall fitness curves between populations. The key summary statistics for these models are given in Table H in S1 Appendix. The lowest CTmin was 17.8°C for the Monterrey population while the highest CTmin was 19.7°C for the Acapulco population. The lowest CTmax was 31.1°C for the Jojulta population while the highest CTmax was 34.2°C for Monterrey. The lowest Topt was 26.1°C for Monterrey and the highest Topt was 28.3°C for Juárez. The population from Monterrey had both the lowest CTmin and the highest CTmax, giving it the greatest thermal breadth of 8.4°C, while the smallest Tbreadth of 6.2°C was observed for Acapulco and Jojutla populations. There were also varying Pmax values (the fitness value at the optimum temperature) with the highest Pmax being 0.504 for Cabo and the lowest Pmax being 0.358 for Jojutla. Pearson’s correlations revealed a positive correlation between fitness Pmax and Topt (r = 0.882), as well as fitness Pmax and Tbreadth (r = 0.861) (Fig 3). Lastly, we used Pearson’s correlation which indicated a strong positive correlation between fitness Topt and the latitude of the home environment (r = 0.863) (Fig E in S1 Appendix).

**Fig 4:**
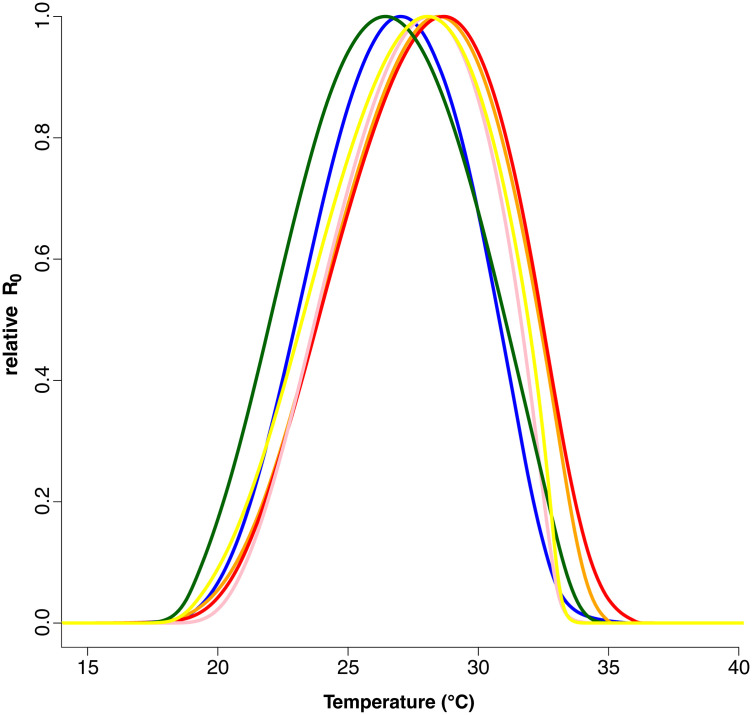
Temperature-dependent relative R_0_ (transmission rate as the basic reproduction rate R_0_) derived from individual life history traits measured for *Ae. aegypti* mosquitoes reared at 13°C, 15°C,19°C, 23°C, 25°C, 27°C, 29°C, 31°C, 33°C, 35°C, 37°C. We measured mosquito life-history traits for five populations from Mexico (Cabo San Lucas (yellow), Acapulco (pink), Monterrey (orange), Ciudad Juárez (red), and Jojutla (blue)) compared to a laboratory-adapted line (shown in green). The lines indicate mean model fits. Fits for individual populations that include the credible intervals and model summary values are presented in Fig D and Table I in S1 Appendix.

Finally, possible implications for transmission are presented in Fig 4, which shows the thermal performance curves for dengue virus transmission potential for each population using relative R_0_ as a comparative metric. The key summary statistics for these models are given in Table I in S1 Appendix. We observe clear differences in the temperature sensitivity of transmission between individual populations. The lowest CTmin was 17.4°C for the Jojutla population and the highest CTmin was 20.2°C for the Acapulco population. The lowest CTmax was 32.8°C for Acapulco and the highest CTmax was 33.7°C for Monterrey. The lowest Topt was 26.1°C for the laboratory adapted population and the highest Topt was 28.6°C for the population from Juárez.

## Discussion

In this study, we used a common-garden laboratory experiment to examine the thermal performance of five field-derived populations of *Ae. aegypti*, together with a longstanding lab strain. This approach, that minimizes the impact of environmental variation, is designed to reveal phenotypic differences across populations due to genetic variation [67,69,70]. Specifically, we measured individual lifehistory traits and created composite metrics for both population fitness and dengue transmission potential. The results reveal significant between-population differences in several life-history traits leading to distinct thermal performance curves for fitness and transmission potential. These results have important implications for understanding the effects of temperature and climate change on the transmission of vector-borne disease.

Examination of individual life-history traits reveals a slightly mixed picture, with large between-population differences in thermal performance of certain traits (e.g., fecundity) but more conserved performance curves for others (e.g., mosquito development rate). Previous studies in a range of other taxa reveal differences in thermal performance curves among traits and life stages [90–94], indicating that thermal responses are not necessarily adapted predictably or uniformly across all traits [95,96]. These studies predicted that the rate of thermal adaptation in an arthropod population is constrained by changes in peak performance temperatures for key life-history traits [96].

Changes in the growth rate of populations are directed by shifts in thermal performance curves of individual traits, which are expected to shift more rapidly than overall fitness with selection because of more thermodynamic constraints [97–99]. To determine overall differences in thermal performance between populations, we therefore combine individual life-history traits into measures of population fitness. This is an important step as most studies do not characterize all traits necessary to estimate fitness [94]. In this study, these fitness models further reveal differences in thermal performance curves between populations (Figs 2 and C and Table H in S1 Appendix).

The physiological and evolutionary factors that shape variation in thermal performance curves have been the subject of much research and center around two main hypotheses. The ‘hotter-is-better’ hypothesis argues that because of thermodynamic constraints, the maximal performance of organisms with high optimal temperatures should be greater than that of organisms with low optimal temperatures and will tend to have greater thermal breadth [48,100–103]. The ‘jack-of-all-temperatures’ hypothesis argues that a trade-off should exist between maximal performance and breadth of performance because different factors of a thermal performance curve can evolve independently and the association between these factors would be shown through evolutionary trade-offs [104–108]. In our fitness curves, the positive correlation between maximum fitness (Pmax) and optimum temperature (Topt), as well as maximum fitness (Pmax) and thermal performance breadth (Tbreadth) (Fig 3) provide no signal of a generalist-specialist trade-off and are more consistent with the ‘hotter-is-better’ hypothesis.

The differences in thermal performance of the individual mosquito populations further lead to differences in temperature-dependent transmission potential of dengue virus (Fig 4). Interpreting these results in terms of absolute transmission risk is not possible as we did not measure infection traits empirically. Instead, we used previously published data for the thermal performance curves of vector competence and pathogen development rate that are not specific to our populations [10]. However, as with other traits, vector competence and pathogen development rate might differ between populations [109,110]. Further, estimating absolute R_0_ requires estimates of the vector-host ratio and we do not have measures of human density in the different locations, nor can we predict absolute vector densities since these could vary due to a range of factors independent of temperature. Nonetheless, the estimates of relative R_0_ enable us to draw several important insights concerning the possible effects of temperature and temperature change on dengue transmission potential with local adaptation of the mosquito vector.

First, transmission potential is expected to differ between populations at particular temperatures. For example, at 29°C the population from Ciudad Juárez is at its maximum transmission potential whereas the population from Jojutla is only at 60%. Conversely, at 25°C the lab and Jojutla populations are at their maximum, whereas Ciudad Juárez is only 60%. Further towards the thermal limits, at 20°C the transmission potential for Acapulco is close to zero yet relative R_0_ for the lab population is 15%, while at 33°C both Cabo San Lucas and Acapulco are close to zero, yet Monterrey and Ciudad Juárez have relative R_0_ values above 40%. While we did not directly measure vector competence traits, we indicate substantial effect sizes and clearly demonstrate that thermal dependence of transmission potential differs between populations and is not well represented by any of the single models. To illustrate this point further, the generic thermal performance curve for relative R_0_ developed by Mordecai et al. (2017) using data from multiple published studies has a Topt of 29.1°C (95% CI: 28.4-29.8°C), a CTmin of 17.8°C (95% CI: 14.6-21.2°C) and CTmax of 34.6°C (95% CI: 34.1-35.6°C) [10]. In our relative R_0_ models we see the Topt of the individual populations ranging from 26.1 (95% CI: 27.1-25.9°C) to 28.4°C (95% CI: 28.3- 28.5°C), CTmin from 17.4 (95% CI: 16.6- 23.0°C) to 20.2°C (95% CI:23.1-18.7°C), and CTmax from 32.7 (95% CI:31.3- 33.7°C) – 36.1°C (95% CI:28.6- 36.9°C). As an additional observation, while our field populations differ from one another, the largest outlier is the long-standing lab strain. Accordingly, our results provide a possible cautionary note to the many studies that explore aspects of transmission of vector-borne diseases using data from lab strains alone. This result might be expected among long-standing lab strains as they are often maintained in constant conditions over many years.

Second, a common approach to explore the possible implications of climate warming is to track changes in transmission across the R_0_ thermal performance curve [7,9–11,27,35–38]. With a single curve, a particular shift in temperature yields a predictable response. However, if thermal response curves differ between individual populations the responses to shifts in temperature will be more idiosyncratic. For example, a warming of 3°C from 26 to 29°C would suggest an increase in relative R_0_ of around 10% for Monterrey but a decline of 30% for Jojutla. A shift from 29 to 32°C would cause a decline in transmission potential in all populations but for Ciudad Juárez the relative R_0_ would still be at 25%, whereas transmission potential for Acapulco would be almost zero.

Third, the standard approach of applying thermal performance curves to predict future changes in transmission due to warming assumes that the thermal performance curves themselves remain static. However, the fact that we see variation in thermal performance between individual populations indicates thermal performance curves are not fixed. So, while short-term changes in temperature might shift transmission along existing thermal performance curves [42], adaptation could modify the thermal responses in the medium- to longer-term [47]. While absolute limits to adaptation will exist [49,111,112] ongoing adaptation to a changing environment could make responses to climate change more dynamic.

Previous research suggests genetic differentiation between discrete populations of *Ae. aegypti* in Mexico sampled across similar spatial scales to ours [113,114]. Despite using a common garden design, we cannot fully rule out the effects of environment on our phenotypic variation in thermal performance. Furthermore, because we examined a relatively small number of mosquito populations, we have limited power to correlate observed differences in thermal performance curves with specific features of the respective home environments. Hence, it is difficult to conclude that the patterns we observe are a result of adaptation to temperature alone. Research on populations of *Drosophila* species and the mosquito *Culex tarsalis* have demonstrated correlations between thermal performance and proxies of environmental temperature such as gradients in latitude or altitude [64,65]. Other research on the mosquito *Nyssorhynchus darlingi* indicates that variation in life history traits between populations results from both genetic differences among localities as well as plastic responses to differences in temperature [68].

Our results expose the difference in reaction norms, or the range of genetic expression in each trait, across experimental temperatures to understand phenotypic plasticity and adaptation [115]. It is likely, therefore, that the phenotypic differences we observe between populations are a consequence, at least in part, of adaptive responses to temperature. Further analysis indicate that fitness thermal optima are strongly correlated to the latitude of the home environment (Fig E in S1 Appendix). These results show that differences in thermal performance between populations are directionally linked to the home environment, not due to random genetic drift or other external forces, but likely evidence of adaptation. Indeed, in previous research we used experimental passage to demonstrate that differences in thermal performance curves could be generated within 10 generations in response to differences in background temperature alone [47]. Whether the differences in this study are due to genetic adaptation, genetic variation in phenotypic plasticity, or a combination of both, does not alter the functional significance of our results. Future studies should integrate new age genomic techniques such as next-generation sequencing technology to understand the genetic differences between populations [67].

Overall, our study demonstrates between-population differences in thermal performance *Ae. aegypti* mosquitoes that influence the predicted effects of temperature on mosquito fitness and dengue transmission potential at the local level. These results add complexities for extrapolating single thermal performance models over space and time, since existing adaptation could result in idiosyncratic responses of individual populations, and future local adaptation could further shift thermal performance curves. Given the public health significance of the multitude of pathogens transmitted by *Aedes* and other mosquitoes (e.g., dengue, Zika, Chikungunya, Yellow Fever, malaria, West Nile, filariasis, Ross River virus) and interests in the influence of climate change, this research highlights the importance of better characterizing the thermal dependence of mosquitoes and their pathogens. More generally, the interacting effects of local adaption and climate change could extend to many systems, including other vector-borne diseases of humans (e.g., Chagas disease transmitted by triatomine bugs [116–118]), animals (e.g., Bluetongue virus transmitted by culicoid midges [119–122]) and plants (e.g., numerous viruses transmitted by Hemiptera [123,124].

## Supporting information

S1 AppendixText A.Quantitative genetics variance decomposition model. **Text B**. Expanded mathematical models supporting the methods. **Fig A. Lifespan fecundity.** Thermal responses for *Aedes aegypti* eggs per female per day for mosquitoes from Mexico compared to a laboratory line. Uninformative priors were used, and the models were fit to average fecundity for each replicate. We measured life history traits for five populations from Mexico (Cabo San Lucas (yellow), Acapulco (pink), Monterrey (orange), Ciudad Juárez (red), and Jojutla (blue)) compared to a laboratory adapted line (shown in green). Each line was tested between 13°C-37°C, but individuals could not survive and reproduce at 13°C and 37°C. **Fig B**. **Juvenile mortality rate thermal performance curve.** Thermal responses for *Aedes aegypti* juvenile mortality rate for mosquitoes from Mexico compared to a laboratory line. Uninformative priors were used, and the models were fit to raw data. We measured life history traits for five populations from Mexico (Cabo San Lucas (yellow), Acapulco (pink), Monterrey (orange), Ciudad Juárez (red), and Jojutla (blue)) compared to a laboratory adapted line (shown in green). Each line was tested between 13°C-37°C, but individuals could not survive and reproduce at 13°C and 37°C. **Fig C. Temperature-dependent fitness models.** Temperature-dependent fitness (intrinsic rate of increase, r_m_) derived from individual life history traits measured for *Ae. aegypti* mosquitoes reared at 13°C, 15°C, 19°C, 23°C, 25°C, 27°C, 29°C, 31°C, 33°C, 35°C, 37°C. We measured life history traits for five populations from Mexico (Cabo San Lucas (yellow), Acapulco (pink), Monterrey (orange), Ciudad Juárez (red), and Jojutla (blue)) compared to a laboratory-adapted line (shown in green). The lines indicate mean model fits while the shaded area indicates the 95% credible intervals. **Fig D. Temperature-dependent relative R**_**0**_ **models.** Temperature-dependent relative R_0_ (transmission rate as the basic reproduction rate R_0_) derived from individual life history traits measured for *Ae. aegypti* mosquitoes reared at 13°C, 15°C,19°C, 23°C, 25°C, 27°C, 29°C, 31°C, 33°C, 35°C, 37°C. We measured mosquito life history traits for five populations from Mexico (Cabo San Lucas (yellow), Acapulco (pink), Monterrey (orange), Ciudad Juárez (red), and Jojutla (blue)) compared to a laboratory-adapted line (shown in green). The lines indicate mean model fits while the shaded area indicates 95% credible intervals. Table A. Information on locations for *Aedes aegypti* mosquito collection in Mexico. Data collected over multiple years in each city. Each mean represents the grand mean across multiple years. These populations were compared to a standard laboratory adapted line (Rockefeller strain) maintained at Penn State at standard insectary conditions. **Table B. Briere model output for mosquito development rate for *Aedes aegypti* from Mexico compared to a laboratory adapted line.** Data for mosquito development rate for five populations of *Ae. aegypti* mosquitoes from Mexico compared to one laboratory adapted line were used to generate briere model. Mean and 95% credible interval (95% HPD interval) for the critical thermal minimum (T_0_), maximum, (T_m_), and a rate constant (*c*) are given for mosquito development rate. The Deviance Criterion Information (DIC) is given to compare model fits. We are comparing this model to one that fits all of the points together giving a DIC of -1069.219. **Table C. Quadratic model output for egg to adult survival for *Aedes aegypti* from Mexico compared to a laboratory adapted line.** Data for egg to adult survival on *Ae. aegypti* from 5 population from Mexico compared to one laboratory adapted line were used to generate quadratic model. Mean and 95% credible interval (95% HPD interval) for the critical thermal minimum (T_0_), maximum, (T_m_), and a rate constant (*c*) are given for egg-to-adult survival. The Deviance Information Criterion (DIC) is given to compare model fits. We are comparing this model to one that fits all of the points together giving a DIC of 6133. **Table D. Quadratic model output for eggs per female per gonotrophic cycle for *Aedes aegypti* from Mexico compared to a laboratory adapted line.** Data for eggs per female per day on *Ae. aegypti* mosquitoes selected over ten generations were used to generate quadratic model. Mean and 95% credible interval (95% HPD interval) for the critical thermal minimum (T_0_), maximum, (T_m_), and a rate constant (*c*) are given for fecundity. The Deviance Criterion Information (DIC) is given to compare model fits. We are comparing this model to one that fits all of the points together giving a DIC of 9027.19. **Table E. Weibull survival model output for adult survival.** Data for adult survival on *Ae. aegypti* mosquitoes from Mexico along with one laboratory population were used to generate a Bayesian Weibull survival model. Mean and 95% credible interval (95% HPD interval) for the observed lifetimes ($y_{i}$) at some particular temperature are drawn from a Weibull distribution with rate parameter (β) and shape parameter (k). The Deviance Information Criterion (DIC) is given to compare model fits. We are comparing this model to one that fits all of the points together giving a DIC of 104,189. **Table F. Briere model output for biting rate for *Aedes aegypti* from Mexico compared to a laboratory adapted line.** Data for eggs per female per day taken from groups of mosquitoes across their lifetime for five populations of *Ae. aegypti* mosquitoes from Mexico compared to one laboratory adapted line were used to generate quadratic model. Mean and 95% credible interval (95% HPD interval) for the critical thermal minimum (T_0_), maximum, (T_m_), and a rate constant (*c*) are given for eggs per female per day. The Deviance Criterion Information (DIC) is given to compare model fits. We are comparing this model to one that fits all of the points together giving a DIC of -3382.774. **Table G. Quadratic model output for juvenile development rate for *Aedes aegypti* from Mexico compared to a laboratory adapted line.** Data for juvenile mortality rate on *Ae. aegypti* mosquitoes from Mexico compared to a laboratory adapted line were used to generate quadratic model that is used in the fitness model. These data are from an approximation using larval survival rate and the amount of time to the adult stage. Mean and 95% credible interval (95% HPD interval) for the critical thermal minimum (T_0_), maximum, (T_m_), and a rate constant (*c*) are given for mosquito development rate. The Deviance Criterion Information (DIC) is given to compare model fits. We are comparing this model to one that fits all of the points together giving a DIC of -320.1. **Table H. Model output for temperature dependent fitness (*r*).** Data for mosquito temperature dependent population fitness on *Ae. aegypti* mosquitoes from Mexico along with one laboratory population were used to generate a composite model from mosquito development rate, eggs per female per gonotrophic cycle, adult survival, and juvenile mortality rate. Mean and 95% credible interval (95% HPD interval) for the critical thermal minimum (CTmin), maximum, (CTmax), optimum (Topt), thermal breadth (Tbreadth) and maximum thermal potential (Pmax) are given for fitness (*r*). We use the best fit model from each individual life history trait, making this the best fit model. **Table I. Temperature-dependent relative R**_**0**_ **(transmission rate as the basic reproduction rate, R**_**0**_**).** Temperature-dependent relative R_0_ (transmission rate as the basic reproduction rate R_0_) derived from individual life history traits measured for *Ae. aegypti* mosquitoes reared at 13°C, 15°C,19°C, 23°C, 25°C, 27°C, 29°C, 31°C, 33°C, 35°C, 37°C. We measured mosquito life history traits for five populations from Mexico (Cabo San Lucas, Acapulco, Monterrey, Ciudad Juárez, and Jojutla) compared to a laboratory-adapted line. Mean and 95% credible interval (95% HPD interval) for the critical thermal minimum (CTmin), maximum, (CTmax), optimum (Topt), thermal breadth (Tbreadth) and maximum thermal potential (Pmax) are given for fitness (*r*). We use the best fit model from each individual life history trait, making this the best fit model.(DOCX)

## References

[1] Brady OJ, Gething PW, Bhatt S, Messina JP, Brownstein JS, Hoen AG, et al. Refining the global spatial limits of dengue virus transmission by evidence-based consensus. PLoS Negl Trop Dis. 2012;6(8):e1760. doi: 10.1371/journal.pntd.0001760 22880140 PMC3413714

[2] Mayer SV, Tesh RB, Vasilakis N. The emergence of arthropod-borne viral diseases: A global prospective on dengue, chikungunya and zika fevers. Acta Trop. 2017;166:155–63. doi: 10.1016/j.actatropica.2016.11.020 27876643 PMC5203945

[3] Teixeira MG, Siqueira JB Jr, Ferreira GLC, Bricks L, Joint G. Epidemiological trends of dengue disease in Brazil (2000-2010): a systematic literature search and analysis. PLoS Negl Trop Dis. 2013;7(12):e2520. doi: 10.1371/journal.pntd.0002520 24386496 PMC3871634

[4] Chen S-C, Hsieh M-H. Modeling the transmission dynamics of dengue fever: implications of temperature effects. Sci Total Environ. 2012;431:385–91. doi: 10.1016/j.scitotenv.2012.05.012 22705874

[5] Lee H, Kim JE, Lee S, Lee CH. Potential effects of climate change on dengue transmission dynamics in Korea. PLoS One. 2018;13(6):e0199205. doi: 10.1371/journal.pone.0199205 29953493 PMC6023222

[6] Thomas MB. Epidemics on the move: Climate change and infectious disease. PLoS Biol. 2020;18(11):e3001013. doi: 10.1371/journal.pbio.3001013 33232329 PMC7685491

[7] Ryan SJ, Carlson CJ, Mordecai EA, Johnson LR. Global expansion and redistribution of Aedes-borne virus transmission risk with climate change. PLoS Negl Trop Dis. 2019;13(3):e0007213. doi: 10.1371/journal.pntd.0007213 30921321 PMC6438455

[8] Aström C, Rocklöv J, Hales S, Béguin A, Louis V, Sauerborn R. Potential distribution of dengue fever under scenarios of climate change and economic development. Ecohealth. 2012;9(4):448–54. doi: 10.1007/s10393-012-0808-0 23408100

[9] Ryan SJ, Carlson CJ, Tesla B, Bonds MH, Ngonghala CN, Mordecai EA, Johnson LR, Murdock CC. Warming temperatures could expose more than 1.3 billion new people to Zika virus risk by 2050. Glob Change Biol. 2021;27(1):84–93. doi: 10.1111/gcb.15384 PMC7756632 33037740

[10] Mordecai EA, Cohen JM, Evans MV, Gudapati P, Johnson LR, Lippi CA, et al. Detecting the impact of temperature on transmission of Zika, dengue, and chikungunya using mechanistic models. PLoS Negl Trop Dis. 2017;11(4):e0005568. doi: 10.1371/journal.pntd.0005568 28448507 PMC5423694

[11] Shocket MS, Verwillow AB, Numazu MG, Slamani H, Cohen JM, El Moustaid F, et al. Transmission of West Nile and five other temperate mosquito-borne viruses peaks at temperatures between 23°C and 26°C. Elife. 2020;9:e58511. doi: 10.7554/eLife.58511 32930091 PMC7492091

[12] Liu-Helmersson J, Quam M, Wilder-Smith A, Stenlund H, Ebi K, Massad E, et al. Climate Change and Aedes Vectors: 21st Century Projections for Dengue Transmission in Europe. EBioMedicine. 2016;7:267–77. doi: 10.1016/j.ebiom.2016.03.046 27322480 PMC4909611

[13] Colón-González FJ, Sewe MO, Tompkins AM, Sjödin H, Casallas A, Rocklöv J, et al. Projecting the risk of mosquito-borne diseases in a warmer and more populated world: a multi-model, multi-scenario intercomparison modelling study. Lancet Planet Health. 2021;5(7):e404–14. doi: 10.1016/S2542-5196(21)00132-7 34245711 PMC8280459

[14] Romanello M, Napoli C di, Green C, Kennard H, Lampard P, Scamman D, et al. The 2023 report of the Lancet Countdown on health and climate change: the imperative for a health-centred response in a world facing irreversible harms. Lancet. 2023;402(10419):2346–94. doi: 10.1016/S0140-6736(23)01859-7 37977174 PMC7616810

[15] Huey RB, Berrigan D. Temperature, demography, and ectotherm fitness. Am Nat. 2001;158(2):204–10. doi: 10.1086/321314 18707349

[16] Angilletta MJ. Thermal Adaptation: A Theoretical and Empirical Synthesis. 2009. Available from: 10.1093/acprof:oso/9780198570875.001.1

[17] Dell AI, Pawar S, Savage VM. Systematic variation in the temperature dependence of physiological and ecological traits. Proc Natl Acad Sci U S A. 2011;108(26):10591–6. doi: 10.1073/pnas.1015178108 21606358 PMC3127911

[18] Delatte H, Gimonneau G, Triboire A, Fontenille D. Influence of temperature on immature development, survival, longevity, fecundity, and gonotrophic cycles of Aedes albopictus, vector of chikungunya and dengue in the Indian Ocean. J Med Entomol. 2009;46(1):33–41. doi: 10.1603/033.046.0105 19198515

[19] Paaijmans KP, Read AF, Thomas MB. Understanding the link between malaria risk and climate. Proc Natl Acad Sci U S A. 2009;106(33):13844–9. doi: 10.1073/pnas.0903423106 19666598 PMC2720408

[20] Paaijmans KP, Blanford S, Bell AS, Blanford JI, Read AF, Thomas MB. Influence of climate on malaria transmission depends on daily temperature variation. Proc Natl Acad Sci U S A. 2010;107(34):15135–9. doi: 10.1073/pnas.1006422107 20696913 PMC2930540

[21] Lambrechts L, Paaijmans KP, Fansiri T, Carrington LB, Kramer LD, Thomas MB, et al. Impact of daily temperature fluctuations on dengue virus transmission by Aedes aegypti. Proc Natl Acad Sci U S A. 2011;108(18):7460–5. doi: 10.1073/pnas.1101377108 21502510 PMC3088608

[22] Paaijmans KP, Heinig RL, Seliga RA, Blanford JI, Blanford S, Murdock CC, et al. Temperature variation makes ectotherms more sensitive to climate change. Glob Chang Biol. 2013;19(8):2373–80. doi: 10.1111/gcb.12240 23630036 PMC3908367

[23] Grigaltchik VS, Webb C, Seebacher F. Temperature modulates the effects of predation and competition on mosquito larvae. Ecol Entomol. 2016;41(6):668–75. doi: 10.1111/een.12339

[24] Shapiro LLM, Whitehead SA, Thomas MB. Quantifying the effects of temperature on mosquito and parasite traits that determine the transmission potential of human malaria. PLoS Biol. 2017;15(10):e2003489. doi: 10.1371/journal.pbio.2003489 29036170 PMC5658182

[25] Villarreal SM, Winokur O, Harrington L. The Impact of Temperature and Body Size on Fundamental Flight Tone Variation in the Mosquito Vector Aedes aegypti (Diptera: Culicidae): Implications for Acoustic Lures. J Med Entomol. 2017;54(5):1116–21. doi: 10.1093/jme/tjx079 28402550 PMC5850351

[26] Reiskind MH, Janairo MS. Tracking Aedes aegypti (Diptera: Culicidae) Larval Behavior Across Development: Effects of Temperature and Nutrients on Individuals’ Foraging Behavior. J Med Entomol. 2018;55(5):1086–92. doi: 10.1093/jme/tjy073 29771372

[27] Tesla B, Demakovsky LR, Mordecai EA, Ryan SJ, Bonds MH, Ngonghala CN, et al. Temperature drives Zika virus transmission: evidence from empirical and mathematical models. Proc Biol Sci. 2018;285(1884):20180795. doi: 10.1098/rspb.2018.0795 30111605 PMC6111177

[28] Waite JL, Suh E, Lynch PA, Thomas MB. Exploring the lower thermal limits for development of the human malaria parasite, Plasmodium falciparum. Biol Lett. 2019;15(6):20190275. doi: 10.1098/rsbl.2019.0275 31238857 PMC6597502

[29] Suh E, Grossman MK, Waite JL, Dennington NL, Sherrard-Smith E, Churcher TS, et al. The influence of feeding behaviour and temperature on the capacity of mosquitoes to transmit malaria. Nat Ecol Evol. 2020;4(7):940–51. doi: 10.1038/s41559-020-1182-x 32367033 PMC7334094

[30] Zouache K, Fontaine A, Vega-Rua A, Mousson L, Thiberge J-M, Lourenco-De-Oliveira R, et al. Three-way interactions between mosquito population, viral strain and temperature underlying chikungunya virus transmission potential. Proc Biol Sci. 2014;281(1792):20141078. doi: 10.1098/rspb.2014.1078 25122228 PMC4150320

[31] Ruybal JE, Kramer LD, Kilpatrick AM. Geographic variation in the response of Culex pipiens life history traits to temperature. Parasit Vectors. 2016;9:116. doi: 10.1186/s13071-016-1402-z 26928181 PMC4772444

[32] Shapiro LLM, Murdock CC, Jacobs GR, Thomas RJ, Thomas MB. Larval food quantity affects the capacity of adult mosquitoes to transmit human malaria. Proc Biol Sci. 2016;283(1834):20160298. doi: 10.1098/rspb.2016.0298 27412284 PMC4947883

[33] Huxley PJ, Murray KA, Pawar S, Cator LJ. The effect of resource limitation on the temperature dependence of mosquito population fitness. Proc Biol Sci. 2021;288(1949):20203217. doi: 10.1098/rspb.2020.3217 33906411 PMC8079993

[34] Huber JH, Childs ML, Caldwell JM, Mordecai EA. Seasonal temperature variation influences climate suitability for dengue, chikungunya, and Zika transmission. PLoS Negl Trop Dis. 2018;12(5):e0006451. doi: 10.1371/journal.pntd.0006451 29746468 PMC5963813

[35] Mordecai EA, Ryan SJ, Caldwell JM, Shah MM, LaBeaud AD. Climate change could shift disease burden from malaria to arboviruses in Africa. Lancet Planet Health. 2020;4(9):e416–23. doi: 10.1016/S2542-5196(20)30178-9 32918887 PMC7490804

[36] Johnson LR, Ben-Horin T, Lafferty KD, McNally A, Mordecai E, Paaijmans KP, et al. Understanding uncertainty in temperature effects on vector-borne disease: a Bayesian approach. Ecology. 2015;96(1):203–13. doi: 10.1890/13-1964.1 26236905

[37] Mordecai EA, Paaijmans KP, Johnson LR, Balzer C, Ben-Horin T, de Moor E, et al. Optimal temperature for malaria transmission is dramatically lower than previously predicted. Ecol Lett. 2013;16(1):22–30. doi: 10.1111/ele.12015 23050931

[38] Caldwell JM, LaBeaud AD, Lambin EF, Stewart-Ibarra AM, Ndenga BA, Mutuku FM, et al. Climate predicts geographic and temporal variation in mosquito-borne disease dynamics on two continents. Nat Commun. 2021;12(1):1233. doi: 10.1038/s41467-021-21496-7 33623008 PMC7902664

[39] Parham PE, Pople D, Christiansen-Jucht C, Lindsay S, Hinsley W, Michael E. Modeling the role of environmental variables on the population dynamics of the malaria vector Anopheles gambiae sensu stricto. Malar J. 2012;11:271. doi: 10.1186/1475-2875-11-271 22877154 PMC3496602

[40] Caminade C, Kovats S, Rocklov J, Tompkins AM, Morse AP, Colón-González FJ, et al. Impact of climate change on global malaria distribution. Proc Natl Acad Sci U S A. 2014;111(9):3286–91. doi: 10.1073/pnas.1302089111 24596427 PMC3948226

[41] Liu-Helmersson J, Stenlund H, Wilder-Smith A, Rocklöv J. Vectorial capacity of Aedes aegypti: effects of temperature and implications for global dengue epidemic potential. PLoS One. 2014;9(3):e89783. doi: 10.1371/journal.pone.0089783 24603439 PMC3946027

[42] Sternberg ED, Thomas MB. Local adaptation to temperature and the implications for vector-borne diseases. Trends Parasitol. 2014;30(3):115–22. doi: 10.1016/j.pt.2013.12.010 24513566

[43] Kingsolver JG, Massie KR, Ragland GJ, Smith MH. Rapid population divergence in thermal reaction norms for an invading species: breaking the temperature-size rule. J Evol Biol. 2007;20(3):892–900. doi: 10.1111/j.1420-9101.2007.01318.x 17465900

[44] Deutsch CA, Tewksbury JJ, Huey RB, Sheldon KS, Ghalambor CK, Haak DC, et al. Impacts of climate warming on terrestrial ectotherms across latitude. Proc Natl Acad Sci U S A. 2008;105(18):6668–72. doi: 10.1073/pnas.0709472105 18458348 PMC2373333

[45] Larson EL, Tinghitella RM, Taylor SA. Insect Hybridization and Climate Change. Front Ecol Evol. 2019;7. doi: 10.3389/fevo.2019.00348

[46] Overgaard J, Sørensen JG. Rapid thermal adaptation during field temperature variations in Drosophila melanogaster. Cryobiology. 2008;56(2):159–62. doi: 10.1016/j.cryobiol.2008.01.001 18295194

[47] Dennington NL, Grossman MK, Ware-Gilmore F, Teeple JL, Johnson LR, Shocket MS, et al. Phenotypic adaptation to temperature in the mosquito vector, Aedes aegypti. Glob Chang Biol. 2024;30(1):e17041. doi: 10.1111/gcb.17041 38273521

[48] Alruiz JM, Peralta-Maraver I, Bozinovic F, Santos M, Rezende EL. Temperature adaptation and its impact on the shape of performance curves in Drosophila populations. Proc Biol Sci. 2023;290(1998):20230507. doi: 10.1098/rspb.2023.0507 37161321 PMC10170199

[49] Kellermann V, van Heerwaarden B, Sgrò CM, Hoffmann AA. Fundamental evolutionary limits in ecological traits drive Drosophila species distributions. Science. 2009;325(5945):1244–6. doi: 10.1126/science.1175443 19729654

[50] Bennett JM, Sunday J, Calosi P, Villalobos F, Martínez B, Molina-Venegas R, et al. The evolution of critical thermal limits of life on Earth. Nat Commun. 2021;12(1):1198. doi: 10.1038/s41467-021-21263-8 33608528 PMC7895938

[51] Weaving H, Terblanche JS, Pottier P, English S. Meta-analysis reveals weak but pervasive plasticity in insect thermal limits. Nat Commun. 2022;13(1):5292. doi: 10.1038/s41467-022-32953-2 36075913 PMC9458737

[52] Couper LI, Farner JE, Caldwell JM, Childs ML, Harris MJ, Kirk DG, et al. How will mosquitoes adapt to climate warming?. Elife. 2021;10:e69630. doi: 10.7554/eLife.69630 34402424 PMC8370766

[53] Abarca M, Parker AL, Larsen EA, Umbanhowar J, Earl C, Guralnick R, et al. How development and survival combine to determine the thermal sensitivity of insects. PLoS One. 2024;19(1):e0291393. doi: 10.1371/journal.pone.0291393 38289939 PMC10826953

[54] Ittonen M, Roberts KT, Lehmann P, Gotthard K. A range‐expanding butterfly is susceptible to cold and long winters but shows no signs of local adaptation to winter conditions. Funct Ecol. 2023;37(12):3064–78. doi: 10.1111/1365-2435.14445

[55] MacLean HJ, Sørensen JG, Kristensen TN, Loeschcke V, Beedholm K, Kellermann V, et al. Evolution and plasticity of thermal performance: an analysis of variation in thermal tolerance and fitness in 22 Drosophila species. Philos Trans R Soc Lond B Biol Sci. 2019;374(1778):20180548. doi: 10.1098/rstb.2018.0548 31203763 PMC6606468

[56] Johansson J. Evolutionary responses to environmental changes: how does competition affect adaptation? Evolution. 2008;62(2):421–35. doi: 10.1111/j.1558-5646.2007.00301.x 18031306

[57] Bürger R, Lynch M. Evolution and extinction in a changing environment: a quantitative-genetic analysis. Evolution. 1995;49(1):151–63. doi: 10.1111/j.1558-5646.1995.tb05967.x 28593664

[58] Lynch M, Lande R. Evolution and extinction in response to environmental change. Biotic Interactions and Global Change. 1993.

[59] Orr HA, Unckless RL. Population extinction and the genetics of adaptation. Am Nat. 2008;172(2):160–9. doi: 10.1086/589460 18662122

[60] Urbanski J, Mogi M, O’Donnell D, DeCotiis M, Toma T, Armbruster P. Rapid adaptive evolution of photoperiodic response during invasion and range expansion across a climatic gradient. Am Nat. 2012;179(4):490–500. doi: 10.1086/664709 22437178

[61] Gatton ML, Chitnis N, Churcher T, Donnelly MJ, Ghani AC, Godfray HCJ, et al. The importance of mosquito behavioural adaptations to malaria control in Africa. Evolution. 2013;67(4):1218–30. doi: 10.1111/evo.12063 23550770 PMC3655544

[62] Egizi A, Fefferman NH, Fonseca DM. Evidence that implicit assumptions of “no evolution” of disease vectors in changing environments can be violated on a rapid timescale. Philos Trans R Soc Lond B Biol Sci. 2015;370(1665):20140136. doi: 10.1098/rstb.2014.0136 25688024 PMC4342969

[63] Ranson H, Lissenden N. Insecticide Resistance in African Anopheles Mosquitoes: A Worsening Situation that Needs Urgent Action to Maintain Malaria Control. Trends Parasitol. 2016;32(3):187–96. doi: 10.1016/j.pt.2015.11.010 26826784

[64] Sgrò CM, Overgaard J, Kristensen TN, Mitchell KA, Cockerell FE, Hoffmann AA. A comprehensive assessment of geographic variation in heat tolerance and hardening capacity in populations of Drosophila melanogaster from eastern Australia. J Evol Biol. 2010;23(11):2484–93. doi: 10.1111/j.1420-9101.2010.02110.x 20874849

[65] Vorhees AS, Gray EM, Bradley TJ. Thermal resistance and performance correlate with climate in populations of a widespread mosquito. Physiol Biochem Zool. 2013;86(1):73–81. doi: 10.1086/668851 23303322

[66] Merilä J, Hendry AP. Climate change, adaptation, and phenotypic plasticity: the problem and the evidence. Evol Appl. 2014;7(1):1–14. doi: 10.1111/eva.12137 24454544 PMC3894893

[67] de Villemereuil P, Gaggiotti OE, Mouterde M, Till-Bottraud I. Common garden experiments in the genomic era: new perspectives and opportunities. Heredity (Edinb). 2016;116(3):249–54. doi: 10.1038/hdy.2015.93 26486610 PMC4806574

[68] Chu VM, Sallum MAM, Moore TE, Lainhart W, Schlichting CD, Conn JE. Regional variation in life history traits and plastic responses to temperature of the major malaria vector Nyssorhynchus darlingi in Brazil. Sci Rep. 2019;9(1):5356. doi: 10.1038/s41598-019-41651-x 30926833 PMC6441093

[69] Savolainen O, Lascoux M, Merilä J. Ecological genomics of local adaptation. Nat Rev Genet. 2013;14(11):807–20. doi: 10.1038/nrg3522 24136507

[70] Kawecki TJ, Ebert D. Conceptual issues in local adaptation. Ecol Lett. 2004;7(12):1225–41. doi: 10.1111/j.1461-0248.2004.00684.x

[71] Napier JD, Heckman RW, Juenger TE. Gene-by-environment interactions in plants: Molecular mechanisms, environmental drivers, and adaptive plasticity. Plant Cell. 2023;35(1):109–24. doi: 10.1093/plcell/koac322 36342220 PMC9806611

[72] Lynch M, Walsh B. Genetics and analysis of quantitative traits Sinauer. Sunderland, MA; 1998.

[73] Falconer DS. Introduction to quantitative genetics. Pearson Education India; 1996.

[74] Wolf JB, Wade MJ. What are maternal effects (and what are they not)? Philos Trans R Soc Lond B Biol Sci. 2009;364(1520):1107–15. doi: 10.1098/rstb.2008.0238 19324615 PMC2666680

[75] Yanchula KZ, Alto BW. Paternal and maternal effects in a mosquito: A bridge for life history transition. J Insect Physiol. 2021;131:104243. doi: 10.1016/j.jinsphys.2021.104243 33845092

[76] Fisher CR, Wilson M, Scott JG. A chromosome-level assembly of the widely used Rockefeller strain of Aedes aegypti, the yellow fever mosquito. G3 (Bethesda). 2022;12(11):jkac242. doi: 10.1093/g3journal/jkac242 36086997 PMC9635639

[77] Dennington N, Thomas M, McGraw E, et al. Phenotypic variation in populations of the mosquito vector, Aedes aegypti, and implications for predicting the effects of temperature and climate change on dengue transmission. [Dataset]. Dryad; 2025. Available from: doi: 10.5061/dryad.sxksn03bm PMC12643283 41252453

[78] Gachohi JM, Njenga MK, Kitala P, Bett B. Modelling Vaccination Strategies against Rift Valley Fever in Livestock in Kenya. PLoS Negl Trop Dis. 2016;10(12):e0005049. doi: 10.1371/journal.pntd.0005049 27973528 PMC5156372

[79] Harrington LC, Fleisher A, Ruiz-Moreno D, Vermeylen F, Wa CV, Poulson RL, et al. Heterogeneous feeding patterns of the dengue vector, Aedes aegypti, on individual human hosts in rural Thailand. PLoS Negl Trop Dis. 2014;8(8):e3048. doi: 10.1371/journal.pntd.0003048 25102306 PMC4125296

[80] Liao C-M, Huang T-L, Cheng Y-H, Chen W-Y, Hsieh N-H, Chen S-C, et al. Assessing dengue infection risk in the southern region of Taiwan: implications for control. Epidemiol Infect. 2015;143(5):1059–72. doi: 10.1017/S0950268814001745 25007831 PMC9507123

[81] Index R, Development TR, Team C. The R Environment for Statistical Computing and Graphics. Vol. 1. Development. 2003.

[82] Plummer M, Best N, Cowles K, Vines K. CODA: convergence diagnosis and output analysis for MCMC. R News. 2006;6(1):7–11.

[83] Su YS, Yajima M. R2jags: a package for running jags from R. R package version 0.03-08.

[84] Plummer M, Best N, Cowles K, Vines K. Coda: Convergence diagnosis and output analysis for MCMC. R News. 2006;6(1).

[85] Amarasekare P, Savage V. A framework for elucidating the temperature dependence of fitness. Am Nat. 2012;179(2):178–91. doi: 10.1086/663677 22218308

[86] Angilletta MJ Jr, Niewiarowski PH, Navas CA. The evolution of thermal physiology in ectotherms. J Therm Biol. 2002;27(4):249–68. doi: 10.1016/s0306-4565(01)00094-8

[87] Spiegelhalter DJ, Best NG, Carlin BP, Van Der Linde A. Bayesian Measures of Model Complexity and Fit. J R Stat Soc Ser B Stat Methodol. 2002;64(4):583–639. doi: 10.1111/1467-9868.00353

[88] Miazgowicz KL, Shocket MS, Ryan SJ, Villena OC, Hall RJ, Owen J, et al. Age influences the thermal suitability of Plasmodium falciparum transmission in the Asian malaria vector Anopheles stephensi. Proc Biol Sci. 2020;287(1931):20201093. doi: 10.1098/rspb.2020.1093 32693720 PMC7423674

[89] Voyles J, Johnson LR, Briggs CJ, Cashins SD, Alford RA, Berger L, et al. Temperature alters reproductive life history patterns in Batrachochytrium dendrobatidis, a lethal pathogen associated with the global loss of amphibians. Ecol Evol. 2012;2(9):2241–9. doi: 10.1002/ece3.334 23139882 PMC3488674

[90] Kingsolver JG, Woods HA. Thermal sensitivity of growth and feeding in Manduca sexta caterpillars. Physiol Zool. 1997;70(6):631–8. doi: 10.1086/515872 9361137

[91] Stevenson RD, Peterson CR, Tsuji JS. The Thermal Dependence of Locomotion, Tongue Flicking, Digestion, and Oxygen Consumption in the Wandering Garter Snake. Physiol Zool. 1985;58(1):46–57. doi: 10.1086/physzool.58.1.30161219

[92] Woods HA, Harrison JF. Interpreting rejections of the beneficial acclimation hypothesis: when is physiological plasticity adaptive? Evolution. 2002;56(9):1863–6. doi: 10.1111/j.0014-3820.2002.tb00201.x 12389732

[93] David JR, Gibert P, Legout H, Pétavy G, Capy P, Moreteau B. Isofemale lines in Drosophila: an empirical approach to quantitative trait analysis in natural populations. Heredity (Edinb). 2005;94(1):3–12. doi: 10.1038/sj.hdy.6800562 15329665

[94] Kellermann V, Chown SL, Schou MF, Aitkenhead I, Janion-Scheepers C, Clemson A, et al. Comparing thermal performance curves across traits: how consistent are they? J Exp Biol. 2019;222(Pt 11):jeb193433. doi: 10.1242/jeb.193433 31085593

[95] Angilletta MJ Jr, Bennett AF, Guderley H, Navas CA, Seebacher F, Wilson RS. Coadaptation: a unifying principle in evolutionary thermal biology. Physiol Biochem Zool. 2006;79(2):282–94. doi: 10.1086/499990 16555188

[96] Pawar S, Huxley PJ, Smallwood TRC, Nesbit ML, Chan AHH, Shocket MS, et al. Variation in temperature of peak trait performance constrains adaptation of arthropod populations to climatic warming. Nat Ecol Evol. 2024;8(3):500–10. doi: 10.1038/s41559-023-02301-8 38273123 PMC10927549

[97] Kingsolver JG. The well-temperatured biologist. (American Society of Naturalists Presidential Address). Am Nat. 2009;174(6):755–68. doi: 10.1086/648310 19857158

[98] Kontopoulos D-G, van Sebille E, Lange M, Yvon-Durocher G, Barraclough TG, Pawar S. Phytoplankton thermal responses adapt in the absence of hard thermodynamic constraints. Evolution. 2020;74(4):775–90. doi: 10.1111/evo.13946 32118294 PMC7384082

[99] Fisher RA. The genetical theory of natural selection. Oxford: Clarendon; 1930.

[100] Bennett AF. Evolution of the control of body temperature: is warmer better. Comparat Physiol. 1987;9:421–31.

[101] Huey RB, Kingsolver JG. Evolution of thermal sensitivity of ectotherm performance. Trends Ecol Evol. 1989;4(5):131–5. doi: 10.1016/0169-5347(89)90211-5 21227334

[102] Savage VM, Gilloly JF, Brown JH, Charnov EL. Effects of body size and temperature on population growth. Am Nat. 2004;163(3):429–41. doi: 10.1086/381872 15026978

[103] Santos M. Evolution of total net fitness in thermal lines: Drosophila subobscura likes it “warm”. J Evol Biol. 2007;20(6):2361–70. doi: 10.1111/j.1420-9101.2007.01408.x 17956397

[104] Montagnes DJS, Wang Q, Lyu Z, Shao C. Evaluating thermal performance of closely related taxa: Support for hotter is not better, but for unexpected reasons. Ecol Monogr. 2022;92(3). doi: 10.1002/ecm.1517

[105] Somero GN, Hochachka PW. Biochemical Adaptation to the Environment. Am Zool. 1971;11(1):159–67. doi: 10.1093/icb/11.1.159

[106] Izem R, Kingsolver JG. Variation in continuous reaction norms: quantifying directions of biological interest. Am Nat. 2005;166(2):277–89. doi: 10.1086/431314 16032579

[107] Gilchrist GW. A quantitative genetic analysis of thermal sensitivity in the locomotor performance curve of Aphidius ervi. Evolution. 1996;50(4):1560–72. doi: 10.1111/j.15585646.1996.tb03928.x 28565727

[108] Huey RB, Hertz PE. Is a Jack-of-All-Temperatures a Master of None? Evolution. 1984;38(2):441. doi: 10.2307/2408502 28555901

[109] Fansiri T, Pongsiri A, Klungthong C, Ponlawat A, Thaisomboonsuk B, Jarman RG, et al. No evidence for local adaptation of dengue viruses to mosquito vector populations in Thailand. Evol Appl. 2016;9(4):608–18. doi: 10.1111/eva.12360 27099625 PMC4831462

[110] Lambrechts L, Chevillon C, Albright RG, Thaisomboonsuk B, Richardson JH, Jarman RG, et al. Genetic specificity and potential for local adaptation between dengue viruses and mosquito vectors. BMC Evol Biol. 2009;9:160. doi: 10.1186/1471-2148-9-160 19589156 PMC2714696

[111] Hoffmann AA, Hallas RJ, Dean JA, Schiffer M. Low potential for climatic stress adaptation in a rainforest Drosophila species. Science. 2003;301(5629):100–2. doi: 10.1126/science.1084296 12843394

[112] Kinzner M-C, Gamisch A, Hoffmann AA, Seifert B, Haider M, Arthofer W, et al. Major range loss predicted from lack of heat adaptability in an alpine Drosophila species. Sci Total Environ. 2019;695:133753. doi: 10.1016/j.scitotenv.2019.133753 31425981

[113] Gloria-Soria A, Ayala D, Bheecarry A, Calderon-Arguedas O, Chadee DD, Chiappero M, et al. Global genetic diversity of Aedes aegypti. Mol Ecol. 2016;25(21):5377–95. doi: 10.1111/mec.13866 27671732 PMC5123671

[114] Gorrochotegui-Escalante N, Munoz ML, Fernandez-Salas I, Beaty BJ, Black WC 4th. Genetic isolation by distance among Aedes aegypti populations along the northeastern coast of Mexico. Am J Trop Med Hyg. 2000;62(2):200–9. doi: 10.4269/ajtmh.2000.62.200 10813474

[115] Oomen RA, Hutchings JA. Genomic reaction norms inform predictions of plastic and adaptive responses to climate change. J Anim Ecol. 2022;91(6):1073–87. doi: 10.1111/1365-2656.13707 35445402 PMC9325537

[116] Carmona-Castro O, Moo-Llanes DA, Ramsey JM. Impact of climate change on vector transmission of Trypanosoma cruzi (Chagas, 1909) in North America. Med Vet Entomol. 2018;32(1):84–101. doi: 10.1111/mve.12269 28887895

[117] Garza M, Feria Arroyo TP, Casillas EA, Sanchez-Cordero V, Rivaldi C-L, Sarkar S. Projected future distributions of vectors of Trypanosoma cruzi in North America under climate change scenarios. PLoS Negl Trop Dis. 2014;8(5):e2818. doi: 10.1371/journal.pntd.0002818 24831117 PMC4022587

[118] Ceccarelli S, Rabinovich JE. Global Climate Change Effects on Venezuela’s Vulnerability to Chagas Disease is Linked to the Geographic Distribution of Five Triatomine Species. J Med Entomol. 2015;52(6):1333–43. doi: 10.1093/jme/tjv119 26336258

[119] Nicolas G, Tisseuil C, Conte A, Allepuz A, Pioz M, Lancelot R, et al. Environmental heterogeneity and variations in the velocity of bluetongue virus spread in six European epidemics. Prev Vet Med. 2018;149:1–9. doi: 10.1016/j.prevetmed.2017.11.005 29290289

[120] Brand SPC, Keeling MJ. The impact of temperature changes on vector-borne disease transmission: Culicoides midges and bluetongue virus. J R Soc Interface. 2017;14(128):20160481. doi: 10.1098/rsif.2016.0481 28298609 PMC5378124

[121] Samy AM, Peterson AT. Climate Change Influences on the Global Potential Distribution of Bluetongue Virus. PLoS One. 2016;11(3):e0150489. doi: 10.1371/journal.pone.0150489 26959424 PMC4784974

[122] Purse BV, Mellor PS, Rogers DJ, Samuel AR, Mertens PPC, Baylis M. Climate change and the recent emergence of bluetongue in Europe. Nat Rev Microbiol. 2005;3(2):171–81. doi: 10.1038/nrmicro1090 15685226

[123] Chen J, Jiang K, Wang S, Li Y, Zhang Y, Tang Z, et al. Climate change impacts on the potential worldwide distribution of the soybean pest, Piezodorus guildinii (Hemiptera: Pentatomidae). J Econ Entomol. 2023;116(3):761–70. doi: 10.1093/jee/toad058 37094809

[124] Skendžić S, Zovko M, Živković IP, Lešić V, Lemić D. The Impact of Climate Change on Agricultural Insect Pests. Insects. 2021;12(5):440. doi: 10.3390/insects12050440 34066138 PMC8150874

